# Nanostructurization of Fe-Ni Alloy

**DOI:** 10.1186/s11671-017-1975-2

**Published:** 2017-03-17

**Authors:** Vitaliy E. Danilhenko

**Affiliations:** 0000 0004 0385 8977 grid.418751.e G.V. Kurdyumov Institute of Metal Physics NAS of Ukraine, Vernadsky Blvd. 36, Kiev, 03680 Ukraine

**Keywords:** Austenite, Martensitic transformation, Nanofragment, Phase hardening, Thermocycling, 68.35.bd Metals and alloys, 68.35.Rh Phase transitions and critical phenomena, 68.37.-d Microscopy of surfaces, interfaces, and thin films

## Abstract

Data about an effect of cyclic γ-α-γ martensitic transformations on the structure state of reverted austenite Fe-31.7 wt.% Ni-0.06 wt.% C alloy are presented. The effect of multiple direct γ-α and reverse α-γ martensitic transformations on fragmentation of austenitic grains has been investigated by electron microscopy and X-ray diffraction methods. An ultrafine structure has been formed by nanofragmentation inside the initial austenite grains due to the successive misorientation of their crystal lattice. Austenite was nanofragmented as a result of multiple γ-α-γ martensitic transformations. Slow heating of the nanofragmented alloy at a rate below 2 °C/s results in nanograin refinement of the structure by multiplication of the reverted γ-phase orientations. The conditions of structure refinement up to ultrafine and nanocrystalline levels as a result of both shear and diffusion mechanisms of reverse α-γ transformation are determined.

## Background

Compacted nanocrystalline metallic materials can be produced by the methods of powder metallurgy and intensive plastic deformation (IPD) [1, 2]. These materials including those having practical importance possess special structure, which provides their unique properties. Nevertheless, the methods mentioned are technologically complicated and energy-consuming. Moreover, the IPD methods are applicable only for thin disc fabrication. These drawbacks are overcome in the approach, which involves refinement of the crystalline structure by the cyclic γ-α-γ (fcc-bcc-fcc) martensitic transformations. The refinement occurs in the process of the reverse α-γ transformation whereby ultrafine or nanocrystalline reverted austenite is formed [3–7]. The structural refinement can be regarded as an extension of the method of austenitic alloy hardening by the reverse α-γ transformations that were firstly developed at Institute of Metal Physics of NASU (Kyiv, Ukraine) and referred to the method of phase hardening [8].

This paper introduces a procedure used for refinement of the Fe-Ni alloy to nanoscale level by the cyclic direct γ-α and reverse α-γ transformations.

## Methods

The subject of this study was metastable Fe-31.7 wt.% Ni-0.06 wt.% C alloy, which was in the austenitic state at room temperature. The direct γ-α transformation occurred when cooling in liquid nitrogen. The reverse α-γ transformation occurred after heating at the rate of 70 °C/s of the samples up to 400 °C in a salt bath (55% NaNO_3_ + 45% KNO_3_). The phase transition kinetics was studied using differential magnetometry [7]. Characteristic temperatures of the γ-α and α-γ transformations were determined by magnetization measurements. The X-ray analysis was carried out on single crystalline samples. To prepare a coarse crystalline ingot in a high-frequency induction furnace, a melt has been slowly cooled down to temperature of 1050 ÷ 1100 °C and then was quenched in salt water. The cylindrical single crystals were cut from coarse grains for the X-ray measurements.

The X-ray diffractometry of single crystalline samples was performed in RCW-86 rotation chamber with photo registration using FeK_α_ radiation. Single crystals were employed to provide an observation of crystal lattice misorientation in the range from a fraction of a degree to several tens of degrees, i.e., to observe the development of the structural fragmentation. The maximum misorientation angle *ψ*, which characterizes the degree of the structural fragmentation, was determined from the azimuthal smearing of the (200)_γ_ and (002)_α_ reflections of the reverted austenite and the martensite. Microstructure was investigated using automatic structure analyser (Epiquant, Germany), transmission electron microscope (TEM) Philips CM-10, and scanning electron microscope (SEM) Jeol JSM-6490-LV (BCE mode).

## Results and Discussion

The ability of austenite to produce the fine structure due to formation of highly dispersed fragments in the process of cyclic martensitic transformations was demonstrated in the work [9]. In this study, austenite recrystallization of nickel-iron alloys after the multiple γ-α-γ transformations (α-γ ones being diffusionless) was firstly described.

**Fig. 1:**
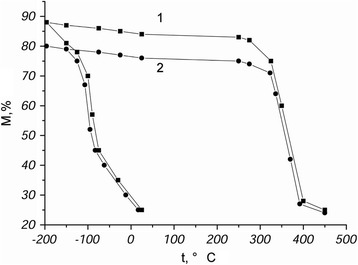
Magnetometric curves of cooling and heating of the alloy in the initial state (*1*) and after 100 γ-α-γ transformations (2). The alloy, wherein a weak stabilization of austenite with respect to the subsequent forward γ-α transformation was observed, has been investigated to reveal the evolution of the structure refinement. During repetitive γ-α-γ transformations, accumulation and interaction of stacking faults had been occurred in austenite that resulted in the formation of additional sub-boundaries and appearance of highly dispersed fragments. Magnetometry showed that amount of martensite in the alloy studied was reduced by only 8% after multiple γ-α-γ transformation cycles

The alloy, in which a weak stabilization of austenite with respect to the subsequent direct γ-α transformation was observed, was investigated to reveal the evolution of the structure refinement. During repetitive γ-α-γ transformations, accumulation, and interaction of stacking faults occurred in austenite and resulted in the formation of additional sub-boundaries and appearance of highly dispersed fragments. Magnetometry showed that amount of martensite in the studied alloy was reduced by only 8% after multiple γ-α-γ transformation cycles (Fig. 1).

The X-ray diffraction pattern of single crystalline specimens demonstrated the azimuthal smearing of the austenite reflections after the reverse α-γ transformation. With increase of the number of cycles, the magnitude of *ψ* was increased and diffraction spots embedded in Debye rings progressively became more smeared along the azimuthal direction. After 80–100 γ-α-γ transformations, the diffraction pattern contained the reflections of the reverted austenite, which was significantly smeared out along the azimuthal direction up to 16°. The γ- and α-phase reflections underwent different extent of azimuthal smearing depending on the degree of crystalline fragmentation. The misorientation of the γ phase was higher than that of the α phase indicating suppression of the martensitic transformation in the most misoriented fragments of the reverted austenite.

**Fig. 2:**
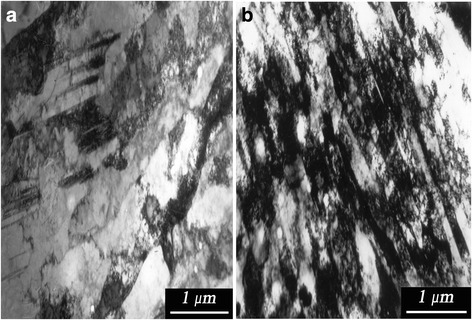
Microstructure of reverted austenite after 3 (**a**) and 100 (**b**) γ-α-γ transformations. The electron microscopy observations have shown that in the reverted austenite, additional sub-boundaries are formed by dislocations introduced during the cyclic γ-α and α-γ martensitic transformations. The dislocation density was found to increase by three orders of magnitude even after the first γ-α-γ cycle. The following cycles hardly raised the dislocation density in line with findings described in [7]. It is important to note that crystalline defects appeared in the process of martensitic transformations are more thermally stable compared to ones formed in plastically deformed alloys. Consequently, they do not vanish within the temperature interval of the reverse transformation and, hence, can accumulate during the following cycling. It is evident that part of dislocations generated during the γ-α and α-γ transformations moves towards sub-boundaries, thus, determining their misorientation angle and size of new fragments. During certain stage of this process, the sub-boundaries form distinct fragments. After 10 cycles, the reflections present in the micrograph are split into 5 ÷ 7 parts that unambiguously indicate the formation of additional sub-boundaries. The sizes of the fragments of the reverted austenite decrease with the number of cycles

The electron microscopy observations have shown that in the reverted austenite, additional sub-boundaries are formed by dislocations introduced during the cyclic γ-α and α-γ martensitic transformations. The dislocation density was found to increase by three orders of magnitude even after the first γ-α-γ cycle. The measuring of dislocation density was fulfilled analogous as in [5]. It is important to note that crystalline defects appeared in the process of martensitic transformations are more thermally stable compared to ones formed in plastically deformed alloys. Consequently, they do not vanish within the temperature interval of the reverse transformation and, hence, can accumulate during following cycling. It is evident that part of dislocations generated during the γ-α and α-γ transformations moves towards sub-boundaries, thus, determining their misorientation angle and size of new fragments. During certain stage of this process, the sub-boundaries form distinct fragments (Fig. 2). After 10 cycles, the reflections present in the micrograph are split into 5 ÷ 7 parts that unambiguously indicates the formation of additional sub-boundaries. The sizes of the fragments of the reverted austenite decrease with the number of cycles. As a result of γ-α-γ transformations, a banded contrast of the same direction which characteristic for deformation twinning was formed. The first signs of the contrast in the structure of reverted austenite appeared after three γ-α-γ transformations (Fig. 2a). After 100 γ-α-γ transformations, the twinning structure of reverted austenite was prevailed (Fig. 2b). The twinning structure appears as a result of accumulation of the inner strains because of volume effect of transformation.

A considerable number of 200–800-nm fragments was observed after 30 cycles. After 60–100 cycles, their sizes were reduced to 70–100 nm.

Our investigations have shown that in nickel-iron alloys, the fragmentation of initial austenite to nanoscale size (nanofragmentation) occurs if the reverse α-γ transformation is diffusionless.

Structural state of the reverted austenite is formed in the process of the multiple reverse diffusionless α-γ transformations and following α-γ transformation controlled by diffusion mechanism. Diffusionless mechanism of reverse α-γ transformation of the investigated alloy occurs at the heating of quenched alloy in the interval of this α-γ transformation with critical rate 70 °C/s. The small amount of carbon up to 0.1 wt.% was not changed of the critical rate of heating.

**Fig. 3:**
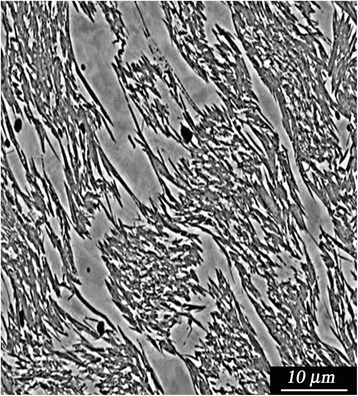
Raster image of a surface of reverted austenite after 100 γ-α-γ transformations with fast heating and one γ-α-γ transformation with slow heating at the interval of α-γ transformation. Special structural state of the reverted austenite is formed in the process of the multiple reverse diffusionless α-γ transformations and following α-γ transformation controlled by diffusion mechanism. Slow heating of the quenched alloy (at the rate of less than 2 °C/s in the interval of the reverse α-γ transformation), which exhibits nanofragmented austenite structure formed during previous 100 γ-α-γ transformations, resulted in the nanograin refinement by multiplication of orientations in the reverted γ phase. The special structural state formed after such a combined treatment, i.e., by using diffusion and diffusionless α-γ transformations, can be characterized by absence of typical for the reverse transformation relief. Instead, the features of plastic deformation of the reverted austenite are seen in figure

Slow heating of the quenched alloy (at the rate of less than 2 °C/s in the interval of the reverse α-γ transformation), which exhibits nanofragmented austenite structure formed during previous 100 γ-α-γ transformations, resulted in the nanograin refinement by multiplication of orientations in the reverted γ phase. The special structural state formed after such a combined treatment, i.e., by using diffusion and diffusionless α-γ transformations, can be characterized by absence of relief, which is typical for the reverse transformation. Instead, the features of plastic deformation of the reverted austenite are seen in Fig. 3.

Additional alloying of Fe-Ni austenite by carbon can increase the grinding degree of reverted austenite in the process of the reverse α-γ -transformation [10–13]. Thermal cycling without γ-α-γ transformations do not result in significant refinment of initial structure [14, 15].

The mechanism involving the formation of highly dispersed nanoscale level structures by cyclic martensitic transformations can be employed for development of metastable alloys with improved physical and mechanical properties.

## Conclusions

The fragmentation of the reverted austenite can occur in the Fe-31.7 wt.% Ni-0.06 wt.% C alloy in the process of the cyclic γ-α-γ transformations due to development of the misorientation processes in the crystal lattice. The cyclic γ-α-γ transformations led to the formation of fragments of nanoscale size; after 80–100 cycles, most of the fragment size was from 70 to 100 nm.

Slow heating of the quenched alloy at a rate below 2 °C/s results in nanograin refinement of the structure by multiplication of the reverted γ-phase orientations. Initially, the nanofragmented structure of the austenite must be formed by the cyclic γ-α-γ transformations. The orientation multiplication takes place due to the diffusion mechanism of the reverse α-γ transformation.

In contrast to the method of the phase hardening, which was earlier used only for hardening, the method of structural refinement by cyclic martensitic transformations is highly promising technique not only for hardening but also for improvement of physical (e.g., magnetic, diffusion, shock-proofing) properties of metastable alloys.

## Abbreviations

Bcc: Body-centered cubic
Fcc: Face-centered cubic
IPD: Intensive plastic deformation
